# Radioresistance, DNA Damage and DNA Repair in Cells With Moderate Overexpression of RPA1

**DOI:** 10.3389/fgene.2020.00855

**Published:** 2020-07-31

**Authors:** Ilya O. Velegzhaninov, Elena S. Belykh, Elena E. Rasova, Yana I. Pylina, Dmitry M. Shadrin, Dmitry Yu. Klokov

**Affiliations:** ^1^Institute of Biology of Komi Scientific Centre of the Ural Branch of the Russian Academy of Sciences, Syktyvkar, Russia; ^2^Institut de Radioprotection et de Sureté Nucléaire, PSE-SANTE, SESANE, LRTOX, Fontenay-aux-Roses, France; ^3^Department of Biochemistry, Microbiology, and Immunology, Faculty of Medicine, University of Ottawa, Ottawa, ON, Canada

**Keywords:** *RPA1* overexpression, CRISPRa, radioresistance, DNA damage, DNA repair

## Abstract

Molecular responses to genotoxic stress, such as ionizing radiation, are intricately complex and involve hundreds of genes. Whether targeted overexpression of an endogenous gene can enhance resistance to ionizing radiation remains to be explored. In the present study we take an advantage of the CRISPR/dCas9 technology to moderately overexpress the *RPA1* gene that encodes a key functional subunit of the replication protein A (RPA). RPA is a highly conserved heterotrimeric single-stranded DNA-binding protein complex involved in DNA replication, recombination, and repair. Dysfunction of RPA1 is detrimental for cells and organisms and can lead to diminished resistance to many stress factors. We demonstrate that HEK293T cells overexpressing *RPA1* exhibit enhanced resistance to cell killing by gamma-radiation. Using the alkali comet assay, we show a remarkable acceleration of DNA breaks rejoining after gamma-irradiation in *RPA1* overexpressing cells. However, the spontaneous rate of DNA damage was also higher in the presence of *RPA1* overexpression, suggesting alterations in the processing of replication errors due to elevated activity of the RPA protein. Additionally, the analysis of the distributions of cells with different levels of DNA damage showed a link between the *RPA1* overexpression and the kinetics of DNA repair within differentially damaged cell subpopulations. Our results provide knew knowledge on DNA damage stress responses and indicate that the concept of enhancing radioresistance by targeted alteration of the expression of a single gene is feasible, however undesired consequences should be considered and evaluated.

## Introduction

Replication protein A (RPA) is a single-stranded DNA-binding protein complex that plays a significant role in maintaining the genome integrity by facilitating DNA replication, recombination, and repair (Audry et al., 2015). RPA was proposed as a first responder at damage sites actively coordinating DNA repair and DNA synthesis (Chen et al., 2016). It modulates the function of DNA helicases, fork remodeling, checkpoint activation and telomere maintenance (Awate and Brosh, 2017). The RPA complex is composed of three subunits of 70, 32, and 14 kDa, called respectively RPA1, RPA2, and RPA3. The complex can bind ssDNA due to its six DNA-binding domains (DBD). DBD-A, -B, -C, and -F are located on the RPA1 subunit, whereas DBD-D and DBD-E are on RPA2 and RPA3, respectively (Maréchal and Zou, 2015). DBD-A, -B, and -C domains are responsible for DNA-protein interaction and DBD-F is located at the N-terminus of the RPA1 subunit and is involved in protein-protein interactions. DBD-F has a crucial role in DNA damage signaling. Indeed, mutations in this part of the protein affecting the conformation disrupted the G2/M checkpoint, inhibited Ddc2/ATRIP and Ddc1/RAD9 interactions and prevented their recruitment to damage sites (Maréchal and Zou, 2015).

In response to DNA damage detected by the XPC-HR23B-CETN2 complex, the RPA1 protein binds to the 5′-end of the undamaged DNA strand opposite the lesion and protects it from nuclease cleavage (Patrick and Turchi, 1999; Oakley, 2010). The RPA complex also plays a key role in the assembly and operation of the repair machinery up to its displacement by polymerases (Oakley, 2010). In homology directed repair of double strand breaks (DSB), RPA1 acts as a factor stabilizing single-stranded DNA ends and prevents the formation of secondary structures (Mimitou and Symington, 2009). Using a cell-free model, the role of the RPA complex in accelerating the non-homologous ends joining (NHEJ) of DNA was also shown (Perrault et al., 2001). Besides its role in several DNA repair pathways (nucleotide excision repair (NER), base excision repair (BER), mismatch repair and DSB repair), RPA proteins support the genome stability by protecting telomeres (Oakley, 2010), as well as by preventing promiscuous annealing between short sequence homologies during replication (Deng et al., 2015).

Although all three RPA proteins are involved in replication, the involvement of RPA1 is not as prominent in comparison to RPA2 and 3. Instead, the RPA1 protein has a more important role in DNA repair. Indeed, mutations of the DBD-A and DBD-B domains of RPA1 lead to a disruption of DNA repair, but had no impact on replication (Hass et al., 2012). Furthermore, it was reported that stable binding and/or melting of secondary DNA structures by RPA1 was required for DNA repair, including RAD51-mediated DNA strand exchange, but was dispensable for DNA replication (Chen et al., 2016).

It is therefore not surprising that a decrease in RPA1 function sensitized cells to ionizing radiation (Dahai et al., 2012), genotoxic chemical agents (Andrews and Turchi, 2004), and heat shock (Fujimoto et al., 2012). Homozygous mutation in the DBD-A domain of RPA1 (Hass et al., 2010) or RNA interferences of *RPA1* gene (Dodson et al., 2004) lead to a disruption of cell cycle progression and DNA repair in HeLa cells. Mice carrying a heterozygous missense change in one of the DBD of RPA1 develop lymphoid tumors, whereas the same homozygous mutation leads to early embryonic lethality (Wang et al., 2005).

High RPA1 expression may serve as a marker of poor prognosis in colon cancer (Givalos et al., 2007), esophageal carcinoma (Dahai et al., 2013) or hepatocellular carcinoma (Wang et al., 2018) patients. In contrast, in bladder urothelial carcinoma the adverse prognosis was inversely correlated with the levels of the RPA1 and RPA2 proteins (Levidou et al., 2011). Adding to this controversy, radioresistance of various nasopharyngeal carcinoma cell lines was shown to correlate with *RPA3*, but not *RPA1* and *RPA2* expression (Qu et al., 2017). However, several authors argue that *RPA1* can be regarded as an oncogene (Wang et al., 2018; Zhu et al., 2018). On the other hand, the important role that RPA1 plays in maintaining the stability of the genome makes this gene a candidate for tumor suppressors. For example, RPA1 may act as a tumor suppressor in the PTEN signaling pathway (Wang et al., 2015). The physical interaction of these two proteins was necessary to protect the replication fork. Consistent with this notion, a heterozygous mutation of *RPA1* promoted tumorigenesis in mice (Wang et al., 2005, 2015).

It can therefore be proposed that overexpression of the *RPA1* gene can positively affect the speed and/or efficiency of DNA repair and can increase cellular radioresistance. The experimental data to this end are scarce and inconsistent. Although an increased resistance to ionizing radiation was demonstrated in the TE-1R cell line with overexpression of *RPA1* (Zhang et al., 2015), since the cell line was generated from irradiated esophageal carcinoma TE-1 cells the radioresistance cannot be conclusively attributed to *RPA1* overexpression. Indeed, it is well known that developing the resistance to ionizing radiation leads to large-scale and extremely complex changes in the transcriptome (Velegzhaninov et al., 2018). Strong simultaneous overexpression of all three RPA subunits in HeLa cells lead to an acceleration of the repair of UV-induced lesions (Bélanger et al., 2016). In contrast, however, it was shown that overexpression of *RPA1* leads to a disruption of homologous recombination and genome instability (Outwin et al., 2011), leaving much controversy whether *RPA1* overexpression can be beneficial for DNA repair and radioresistance.

In the present work, we overexpressed the *RPA1* gene in its natural chromosome context, taking into account all splice variants, using the CRISPRa technology that utilizes the VPR activator fused with the nuclease-null RNA guided protein dCas9 (Chavez et al., 2015; Najm et al., 2018). Cells with transient overexpression were treated with acute gamma-radiation and the survival rate and the rate of DNA repair were analyzed.

## Materials and Methods

### Cells and Plasmids

The experiments were performed using the HEK293T cell line. The cells were maintained in Opti-MEM medium (Gibco, Thermo Fisher Scientific, United States) supplemented with 5% fetal bovine serum (HyClone, Thermo Scientific, United States) without antibiotics at 37°C in a 5% CO_2_ and 95% air atmosphere. For dCas9-VPR expression, the pXPR_120 plasmid was used that was a gift from John Doench & David Root (Addgene plasmid # 96917) (Najm et al., 2018). Oligonucleotides coding sgRNA were cloned into the gRNA Cloning Vector *Bbs*I ver. 2 that was a gift from Hodaka Fujii (Addgene plasmid # 85586) (Fujita et al., 2016).

### sgRNA Design and Cloning

Sequences of sgRNA targeting the promoter of the *RPA1* gene (1–400 nucleotides upstream of the transcription start site) were designed and selected using the Casdesigner online tool (Bae et al., 2014; Table 1). The specificity of sgRNA binding was checked using the Casoffinder online tool (Park et al., 2015). Only those sequences that could not bind to any other target in the human genome, even with two mismatches allowed, were selected. These were further filtered by examining unspecific targeting upon three mismatches allowed and only those that required mismatches in the PAM-proximal part of the sequence were selected. This rule was used because it is known that the specificity of sgRNA at the PAM-proximal end is more important than at the distal end (Hsu et al., 2013). The designed oligonucleotides for cloning in the sgRNA Cloning Vector *Bbs*I ver. Two were synthesized by Evrogen company (Russia). Cloning was performed using the restriction enzyme *Bbs*I-HF (New England Biolabs, United States) and the T4 ligase (Evrogen, Russia).

**TABLE 1:** The sequences of sgRNA targeting the *RPA1* promoter.

Position relative to transcription start site (b.p.)	Sequence 5′–3′
−50	GCGCTACGCAGCCGCCGCAT
−218	GCGCGTCTGAGCGGTTCTCG
−434	GGCCGGGTCTGATTCCCTTT

### Transfection and Irradiation

Transfection was performed in a 24-well plate using Lipofectamine 3000 (Invitrogen, United States) according to the manufacturer’s protocol. Five hundred nanograms of the pXPR120 plasmid and 500 ng of the sgRNA plasmid mixture was used per one well. The efficiency of transfection was >80% as controlled by co-transfecting with the eGFP expressing LeGO-G2 vector [a gift from Boris Fehse (Addgene plasmid #25917)] (Weber et al., 2008). Forty-eight hours after transfection, the cells were trypsinized and transferred to 12-well plates for the clonogenic survival assay (50 or 200 cells/well) and for the assessment of the proliferation rate. In a separate experiment, after the same transfection protocol, cells were transferred to 96-well plates for the analysis of survivability by the fluorometric microculture cytotoxicity assay (FMCA, 2000 cells/well). An aliquot of cell suspension at this point was also used for RNA extraction in both experiments, and additionally for protein extraction in the first experiment. Cells were allowed to adhere to cell culture plastic surfaces for 4 h and then irradiated with 1, 2, 3, 4, or 6 Gy of gamma-radiation (^137^Cs, 0.74 Gy/min) for the FMCA or 3 Gy only for the clonogenic survival assay.

### Analysis of Survival and Proliferation

Radioresistance was estimated using two different methods and in two independent experiments separated in time. In the first one, the conventional clonogenic survival assay (Puck and Marcus, 1956; Rafehi et al., 2011) was used, whereas the FMCA that measures a fraction of surviving cells (Lindhagen et al., 2008) was used in the second experiment. The survival of cells with or without overexpression of *RPA1* was analyzed 72 h after irradiation using the FMCA (Lindhagen et al., 2008). The results were expressed as mean fluorescence of 24 replicates (microcultures in separate wells/dishes) relative to the mean value of 24 replicates of untreated control. Each transfection group had its own untreated control. The Student’s *t*-test with Bonferroni correction or factorial ANOVA were used for comparison between groups.

For the clonogenic survival assay, cells plated onto 12-well plates were fixed 7 days after irradiation and the number of surviving colonies (>100 cells per colony) was scored. The results were expressed as mean number of colonies in 12 replicate wells relative to untreated control. For assessing the proliferation rate, 25 colonies were randomly selected from each treatment/transfection group and the number of cells per colony was counted. Each experiment and plating format had its own control plated from the same original cell suspension. For both readouts, the Student’s *t*-test was used for comparison between groups.

### qRT-PCR

Total RNA was extracted using the Aurum Total RNA Mini Kit (BioRad, United States) as per manufacturer’s instructions. Extracted RNA was quantified using the Qubit^TM^ RNA BR Assay Kit and a Qubit^TM^ fluorometer (Thermo Fisher Scientific, United States). One microgram of total RNA per sample was reverse transcribed into cDNA using the Maxima First Strand cDNA Synthesis Kit (Thermo Fisher Scientific, United States) as per manufacturer’s recommendations. The real time PCR reactions were conducted using qPCRmix-HS SYBR (Evrogen, Russia) on a CFX96 PCR Detection System (Bio-Rad, United States). The following PCR cycling conditions were used: 95°C for 5 min, 40 cycles of 95°C for 15 s, 58°C for 15 s and 72°C 30 s. Each analysis was carried out in three technical replicates. Relative expression was calculated using the ΔΔCt method (Livak and Schmittgen, 2001) by normalizing to the house keeping genes *ACTB* and *GAPDH*. Data were analyzed using the CFX Manager (Bio-Rad, United States) and Excel (Microsoft, United States) software. Primers for *RPA1* were designed using Primer-BLAST online tool (Ye et al., 2012) (forward-AAGGCACCCTGAAGATTGCT, reverse-CAGGGCATGACGGAAGTCTC). Primer sequences for *GAPDH* were taken from Cheng et al. (2007) (forward-ACACCCACTCCTCCACCTTTG, reverse-GCTGTAGCCAAA TTCGTTGTCATAC), and for *ACTB* from Ding et al. (2009) (forward-GCGCGGCTACAGCTTCA, reverse-CTTAATGTCA CGCACGATTTCC). Oligonucleotides were synthesized by Evrogen (Russia).

### Comet Assay

DNA damage was evaluated using the alkaline comet assay that detects DNA single strand breaks, DSB and alkali-labile sites (Tice et al., 2000). The cells were detached by 50 μL 0.05% Trypsin-EDTA solution with Hanks salts (PanEco, Russia) and then mixed with 1200 μL of Opti-MEM medium. The resulting suspension was processed differently for collecting the initial time point of 1 min post-irradiation vs. 5, 30, and 60 min post-irradiation. For the initial time point of 1 min, cells were immobilized in low melting point agarose (prepared in PBS at pH 7.4 and 37.5°C) on slides and then irradiated at 3 Gy followed by immediate fixation in the lysis solution (2.5 M NaCl, 100 mM Na_2_EDTA, 10 mM Tris-HCl, pH 10.0, 10% DMSO, 1% Triton X-100). For 5, 30, and 60 min time points, cells were irradiated in suspension, then incubated at 37°C for corresponding periods of time and then rapidly immobilized in agarose gel followed by fast cooling on a 4°C surface and immersing to the lysis solution. Cells were lysed overnight at 4°C and then incubated in the alkaline electrophoresis buffer (300 mM NaOH, 1 mM EDTA, pH > 13) for 40 min at 4°C for DNA unwinding. Next, the slides were subjected to electrophoresis at 1 V/cm, 300 mA at 4°C for 25 min. Following an extensive rinse in the neutralizing buffer (0.1 M Tris–HCl, pH 7.5) and then in bi-distilled water for 15 min, both at 4°C, the cells were fixed in ethanol for 10 min. The slides were then dried and 100 μL of 2 μg/mL ethidium bromide solution was added to the slides (Sigma-Aldrich). Cover slips were mounted on the slides and sealed with nail polish. The resulting comets were visualized using a fluorescence microscope Axioscop-A1 (Carl Zeiss, Jena, Germany) at 200× magnification. Images were captured using a CCD camera AxioCam ICm 1 and an AxioVision software package (Carl Zeiss) at a 1338 × 1038 pixels resolution. Percent DNA in comet tail (%DNA) was calculated using the CometScore Pro software (TriTek Corp, United States). The mean value of %DNA from 100 comets per slide was calculated and used as an index of DNA damage. Nine slides (three slides from each of three technical replicates of cell suspensions) were analyzed for each experimental group. Median %DNA was calculated from the nine slides per group and was used as an integral measure of the level of DNA damage per group. All scored cells were used to generate a distribution of cells containing various levels of DNA damage. Differences in the distributions of cells with% DNA in Coment tail (in the range of 0–49) were analyzed by the Pearson χ^2^-test.

### Western Blotting

Cells were lysed in RIPA buffer (20 mM Tris pH 7.5, 150 mM NaCl, 1 mM EDTA, 1% NP-40, 0.5% sodium deoxycholate, 0.5% Sodium Dodecyl Sulfate containing protease and phosphatase inhibitors (Roche, Germany) 48 h after transfection with pXPR_120 and *Bbs*I_CV plasmids with or without sgRNAs to *RPA1* promotor. Total protein concentrations in the lysates were determined using the Quick Start^TM^ Bradford Protein Assay Kit (Biorad, United States) by measuring absorption at 595 nm on a Fluorat-02 Panorama spectrophotometer (Lumex, Russia). For each sample, a total of 30 μg of protein was resolved on 10% acrylamide gels (TGX FastCast Acrylamide Kit, Biorad, United States) and transferred to PVDF membranes (Bio-Rad). After blocking with 3% bovine serum albumin (BSA) in TBST (0.1% Tween-20, 150 mM NaCl, 20 mM Tris, pH 7.5) for 1 h, the membranes were incubated overnight at 4°C with the primary antibodies (Rabbit polyclonal Anti-RPA70 antibody (ab12320, Abcam, United Kingdom) diluted in TBST with 3% BSA. After washing in TBST, the membranes were incubated for 1 h at room temperature with recombinant anti-rabbit IgG VHH single domain (HRP) (ab191866) antibody (Abcam, United Kingdom). Following three rinses in TBS, the Immun-Star Western C reagent (Bio-Rad) was used to initiate chemiluminescence and the signal was imaged using a Chemidoc XRS imager (Bio-Rad). Quantification of the signal from recorded images was performed using the ImageLab (Bio-Rad) software.

## Results and Discussion

**FIGURE 1:**
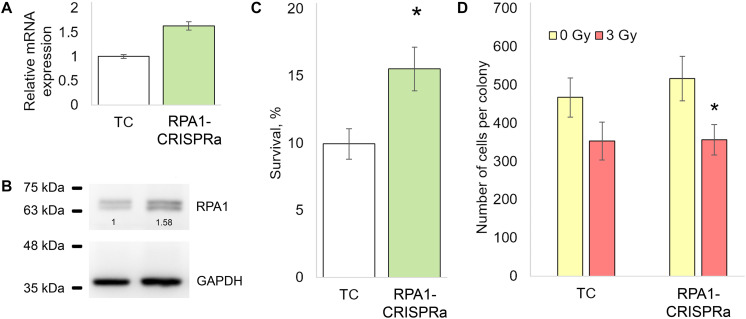
The effect of the overexpression of the *RPA1* gene on the resistance of HEK293T cells to ionizing radiation and the proliferation rate. **(A,B)** The levels of mRNA (qRT-PCR) and protein (western blotting) of *RPA1* 48 h after cotransfection of cells with the plasmids encoding the dCas9-VPR activator and guide RNAs (three biological replicates per group in each analysis). **(C)** The proportion of surviving CFU after exposure to γ-radiation at 3 Gy (12 replicates per group). **(D)** The average number of cells per coloniy (25 randomly selected colonies per group). *, differences with the TC group are significant at *p* < 0.05 (Student’s *t*-test).

We first examined the effectiveness of the CRISPRa/dCas9-VPR system by measuring the *RPA1* mRNA levels in cells transfected with dCas9-VPR and *RPA1* sgRNAs. A total of 48 h post-transfection, a 1.5-fold increase in the *RPA1* mRNA level was found compared to cells transfected with empty plasmids (Figure 1A). Using western blotting, we further showed that this mRNA overexpression did result in higher RPA1 protein levels (Figure 1B). Importantly, both mRNA and protein increases were consistent at about 1.5-fold change, which represents relatively moderate activation. It is known that the magnitude of overexpression by CRISPRa negatively correlates with the basal expression level of a target gene (Chavez et al., 2015; Konermann et al., 2015). Therefore from a technical point of view, the moderate activation of *RPA1* achieved in our study was not an unexpected result. To examine how this overexpression of *RPA1* altered radiosensitivity, an aliquot of the transfected cells, together with empty (TC) and *PRA1* targeting *Bbs*I_CV plasmid, were seeded on 12-well plates, exposed to sham- or a gamma-radiation dose of 3 Gy and the clonogenic survival was measured. We observed that the *RPA1* overexpression resulted in a 50% higher radioresistance compared to the cells without the altered *RPA1* levels (Figure 1C).

Since proteins of the RPA family play a key role in DNA replication (Audry et al., 2015), we examined whether the higher number of surviving colonies in *RPA1* overexpressing cells may have been caused by accelerated proliferation rather than damage removal. To this end, we scored the number of cells in randomly selected surviving colonies in both TC and RPA1-CRISPRa cells. We found no difference between these groups suggesting that proliferation was not affected by the *RPA1* overexpression and played no role in the observed radioresistance (Figure 1D).

**FIGURE 2:**
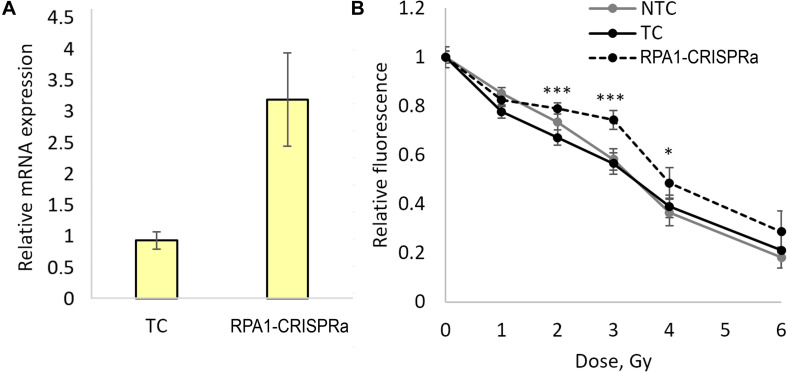
Overexpression of the *RPA1* gene detected using qRT-PCR (three samples analyzed per group) **(A)** and cell viability 72 h after exposure to γ-radiation at 1, 2, 3, 4, and 6 Gy analyzed using the FMCA **(B)**. Average values for 24 microcultures per data point are presented. Relative fluorescence is proportional to the number of live cells in the microculture. *, differences with the TC group are significant at *p* < 0.05, *** at *p* < 0.001 (Student’s *t*-test with the Bonferroni correction).

In order to validate these findings and examine the responses in a broader dose-range, we carried out another series of transfections and achieved a threefold induction of *RPA1* (Figure 2A). As in experiments above, aliquots of the transfected cells were used for examining radioresistance. Cells were seeded onto 96-well plates 48 h after transfection and irradiated with five different doses of gamma-radiation, 1, 2, 3, 4, and 6 Gy. A total of 72 h after irradiation, the relative number of surviving cells was measured using the FMCA and plotted as survival curves (Figure 2B). The results indicate a moderate but significant and reproducible increase in a resistance to radiation-induced cell killing at doses of 2, 3, and 4 Gy in cells overexpressing *RPA1*. Furthermore, the comparison of the FMCA survival curves for TC and CRISPRa-RPA1 groups using the factorial ANOVA produced a highly significant difference (*p* < 0.000001).

**FIGURE 3:**
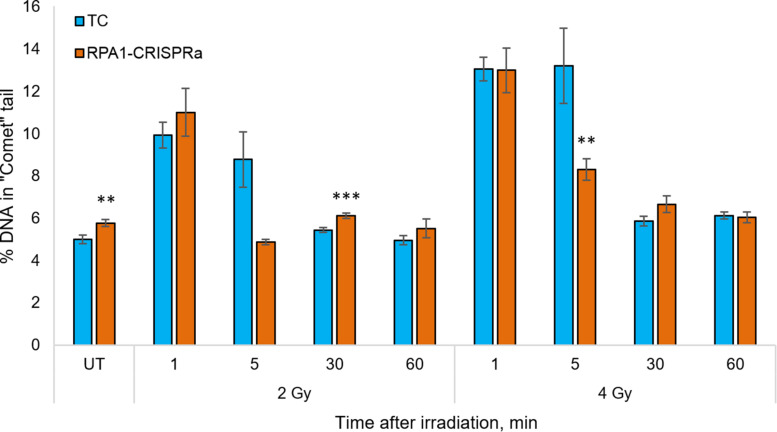
The level of DNA damage in HEK293T cells at 1, 5, 30, and 60 min after irradiation with 2 and 4 Gy. The median value from more than 100 cells per slide were calculated and the mean per nine slides is presented for each group/time point. ** and *** denote statistically significant difference vs. the TC group at *p* < 0.01 and 0.001, respectively (Student’s *t*-test).

Since the RPA1 plays a substantial role in DNA repair and maintaining the genome stability, and since we showed that its overexpression renders cells radioresistant in a manner independent of proliferation (Figure 1D), we next analyzed the DNA repair rates in cells with overexpressed *RPA1* compared to TC. The analysis was performed using an alkaline version of the comet assay in cells irradiated with 2 or 4 Gy at time-points of 0 (UT), 1, 5, 30, and 60 min post-exposure (Figure 3). First, we found a small but statistically significant increase in the basal level of DNA damage (Figure 3, group UT). This somewhat surprising observation may be explained by the function of RPA1 in correcting replication errors. It is feasible to suggest that proper processing of these spontaneous DNA lesions that depends on timing of RPA1 binding and dissociation from damaged single-stranded DNA and on the equilibrium between RPA1 and other DNA repair factors may be altered upon elevated RPA1 levels. Previously, the induction of genomic instability by overexpression of *RPA1* was described by Outwin et al. (2011). The authors hypothesized that ectopic expression of *RPA1* affects homologous recombination pathways.

Previous reports (for example (Velegzhaninov et al., 2015) among many others) indicated that in human cells, all single-stranded radiation-induced DNA damage is repaired within the first few minutes. In the present study we also observed that both in the control cells and *RPA1* overexpressing cells the radiation-induced DNA damages was not measurable from 30 min post-irradiation. However, at 5 min after 4 Gy there was a lower level of DNA damage in *RPA1* overexpressing cells compared to TC. For 2 Gy, a very similar response was seen, however with no statistical significance, which is most likely attributable to the large variability of electrophoretic mobility of DNA in freshly irradiated cells. The alkaline comet assay detects a totality of single and double-stranded DNA breaks (SSB and DSB), as well as alkali-labile sites (Tice et al., 2000). However, we suggest that the overall immediate repair rate, detected by the alkali comet assay and the observed increase in the repair rate in RPA1 overexpressing cells occurs mainly due to the SSB component. Our assumption is based on two facts. First, gamma-radiation causes many times more SSB than DSB (Nikjoo et al., 1999; Watanabe et al., 2015). Secondly, the repair rate of SSB (Sweigert et al., 1989; Churchill et al., 1991; Rahmanian et al., 2014; Velegzhaninov et al., 2015) is higher, or at least same as that of DSB by NHEJ (Metzger and Iliakis, 1991; Núñez et al., 1995; DiBiase et al., 2000; Stenerlöw et al., 2003; Wang et al., 2006). Furthermore, it is feasible to suggest that the enhanced repair of SSB could affect cellular radioresistance due to a more efficient elimination of clustered DNA lesions. Such damage is poorly repaired, can turn into DSB and at the same time clustered damage sites are 3–4 times more frequent upon gamma-radiation than the DSB itself (Georgakilas et al., 2013; Sage and Shikazono, 2017). It is believed that the repair of clustered non-DSB damage requires increased activity of the long patch BER mechanism (Bukowska and Karwowski, 2018), which is known to be stimulated by the RPA complex (DeMott et al., 1998).

**FIGURE 4:**
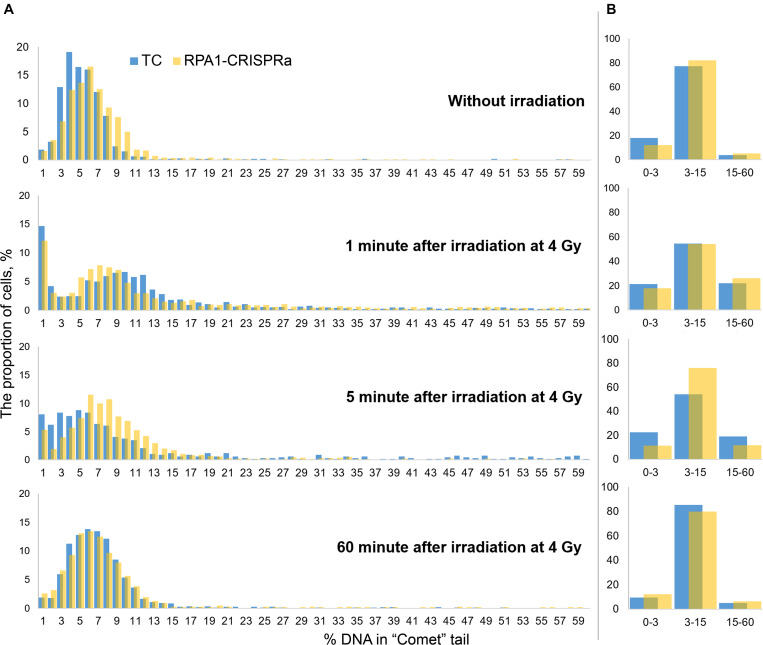
Histograms of the distribution of DNA damage at various time points after irradiation. Cells were pooled either into 1% increments of %DNA in tail **(A)** for detailed histograms or into three categories of low (1–3 %DNA), intermediate (3–15 %DNA) and high (15–60 %DNA) level of electrophoretic mobility **(B)**. In **(A)**, all nucleoids were analyzed per group. The nucleoids with %DNA more than 60 were not used in the analysis.

Since the comet assay is a single-cell based assay, it provides additional analysis power of examining histograms of the distribution of DNA damage (here, %DNA in tail). Therefore, to better understand the alterations in the repair kinetics due to the *RPA1* overexpression, we draw such distribution histograms with a small DNA damage increment. For this, we combined the data from all replicates within each experiment and binned cells (from 1200 to 1800 cells per treatment group) within 1% (DNA in tail) increments. Resulting distribution histograms are shown in Figure 4A. We also calculated the number of nucleoids with very low (1–3% DNA in “comet” tail), intermediate (3–15%) and high electrophoretic mobility (15–60%) (Figure 4B). Without irradiation, the distribution was close to normal with a center of about 4–5% in the transfection control and about 6% in cultures with transient overexpression of *RPA1*. The Pearson χ^2^-test showed, that these two distribution were different (χ^2^ = 154.75; *p* < 0.000001). Immediately after irradiation, the bulk of the nucleoids shifted to the right, reflecting higher electrophoretic mobility of DNA which can be interpreted as an increase in the amount of DNA damage. After 5 min, as a result of DNA repair, the distributions shifted back to the left. It is interestingly that this shift to the left was more pronounced in TC cells compared to the cells overexpressing *RPA1*. However, the proportion of cells with a high %DNA in tail was significantly lower in RPA1-CRISPRa cells vs. control (Figure 4B), explaining the overall reduction in DNA damage seen in Figure 3. Moreover, the frequency distribution analysis showed more dissimilarity between the distributions at 5 min post-irradiation and without irradiation in TC cells (χ^2^ = 1136.9; *p* < 0.001) as compared to the *RPA1* overexpressing cells (χ^2^ = 283.106; *p* < 0.001), suggesting a faster return to pre-irradiation distribution in *RPA1* overexpressing cells. An important assumption could be made that *RPA1* overexpression leads to faster DNA repair in a subpopulation of high damaged cells. A total of 60 min after irradiation, the distributions were close to normal and almost identical for both TC and the RPA1 overexpressing experimental groups (χ^2^ = 45.2; *p* = 0.59).

It is very interesting that a significant proportion of cells (12–15%) with the extremely low DNA electrophoretic mobility had appeared immediately after irradiation (Figure 4A, 1 min after irradiation). One explanation that seems feasible and very important for interpreting not only this, but any comet assay data, is that these cells are actually cells with radiation-induced DNA interstrand cross-links (Dextraze et al., 2010) and/or DNA-protein cross-links (Merk et al., 2000; Nakano et al., 2015, 2017). This therefore may be indicative of more, not less, damage. With this in mind, it seems that overexpression of *RPA1* can facilitate the repair of these types of lesions since a corresponding decrease in the frequencies of such cells was observed upon *RPA1* overexpression at 5 min post-irradiation (Figure 4B, 5 min after irradiation). Although detailed mechanisms of the repair of DNA-protein crosslinks have not been fully understood (Ide et al., 2011; Fielden et al., 2018), it is known that NER affects the repair efficiency of this type of DNA lesions in the case of cross-linking with small peptides (Nakano et al., 2007), providing support to the suggested interpretation of our results. Interestingly, the presence of low electrophoretic mobility nucleoids after damage induction similar to the one observed in this study was reported by other authors; however, no explanations have been offered (Braafladt et al., 2016). Lastly, as a consequence of the induction of cells with low electrophoretic mobility, we observed a sharp increase in the variance of the mean values (Figure 3) affecting statistical significance of comparing the mean DNA damage values.

The potential of RPA1 to alter cellular radioresistance can be considered within the two biologically distinct contexts: in cancer cells, relevant to radiotherapy, and in normal cells, relevant to the development of radiation toxicity in normal tissues. However, the perspective of the use of *RPA1* overexpression to control the stability of normal cells/tissues remains questionable. Indeed, the concern is related to the increase in the basal level of DNA damage observed in this work, as well as the alterations in homologous recombination and the genome stability previously discovered by Outwin et al. (2011). At the same time, given the effect of the *RPA1* overexpression on the rate of early repair after genotoxic exposure, as well as on the overall radioresistance, further studies of the mechanistic link between the endogenous gene overexpression and the phenotype (cell survival and genome stability) are warranted. The CRISPRa technology appears to be a very useful tool in such research due to its ability to control the expression level (Braun et al., 2016). In addition, a short-term inducible overexpression of the *RPA1* gene limited to the duration of a genotoxic stress may be another attractive possibility that can motivate further research.

Substantially more information is available on the role of RPA1 in tumor resistance to therapeutic genotoxic treatment. *RPA1* expression may be associated with both poor and good prognosis in cancer patients (Givalos et al., 2007; Levidou et al., 2011; Dahai et al., 2013; Wang et al., 2018). Prognosis in cancer treatment is however not necessarily relevant to radioresistance which was the focus of our study. To examine whether RPA1 correlates with radioresistance, we analyzed 16 randomly selected transcriptomics studies where authors used radioresistant vs. radiosensitive cancer cells *in vitro* or cancer biopsies (Achary et al., 2000; Fukuda et al., 2004; Harima et al., 2004; Khodarev et al., 2004; Guo et al., 2005; Hellman et al., 2005; Ogawa et al., 2006, 2008; Xu et al., 2008; Du et al., 2009; Ducray et al., 2010; Souchek et al., 2014; Zhou et al., 2017; Doan et al., 2018; Kim et al., 2018; You et al., 2019). In only one of these studies, the most radiosensitive cell line had a significantly decreased expression of the *RPA1* gene (Ogawa et al., 2008). In contrast, Hellman et al. (2005) reported a decrease in the expression of this gene in radioresistant cells. In all other studies, *RPA1* was not listed as a differentially expressed gene.

Thus, in the present study, we demonstrate that moderate overexpression of *RPA1* in HEK293T cells can increase their radioresistance. This change was low in magnitude and accompanied by a small increase in spontaneous DNA damage rates. At the same time, we found a faster repair of DNA damage in cells with the *RPA1* overexpression, which may be one of the factors contributing to the observed radioresistance. Given the low magnitude of the alterations in the radioresistance as a result of the *RPA1* overexpression observed in this study and in literature, it is feasible to suggest that other genes/mechanisms may have a higher impact on the development of cancer radioresistance. This can be driven by microevolutionary processes favoring selection of the fittest cells. Lastly, our results provide knowledge on the link between *RPA1* expression and the kinetics of DNA repair within differentially damaged cell subpopulations.

## Data Availability Statement

The raw data supporting the conclusions of this article will be made available by the authors, without undue reservation.

## Author Contributions

IV contributed to conception and design of the study. EB, IV, ER, YP, and DS performed the experimental work. IV and DK performed the statistical analysis and wrote the manuscript. All authors contributed to manuscript revision, read, and approved the submitted version.

## Conflict of Interest

The authors declare that the research was conducted in the absence of any commercial or financial relationships that could be construed as a potential conflict of interest.
